# Effects of Estrogen Receptor and Wnt Signaling Activation on Mechanically Induced Bone Formation in a Mouse Model of Postmenopausal Bone Loss

**DOI:** 10.3390/ijms21218301

**Published:** 2020-11-05

**Authors:** Astrid Liedert, Claudia Nemitz, Melanie Haffner-Luntzer, Fabian Schick, Franz Jakob, Anita Ignatius

**Affiliations:** 1 Institute of Orhopedic Research and Biomechanics, Trauma Research Center Ulm, University Medical Center Ulm, 89081 Ulm, Germany; c.nemitz@gmx.net (C.N.); melanie.haffner-luntzer@uni-ulm.de (M.H.-L.); fabian.schick@mailbox.org (F.S.); anita.ignatius@uni-ulm.de (A.I.); 2 Orthopaedic Center for Musculoskeletal Research, University of Würzburg, 97074 Würzburg, Germany; f-jakob.klh@uni-wuerzburg.de

**Keywords:** bone remodeling, mechanotransduction, ER signaling, Wnt/β-catenin signaling, ovariectomy

## Abstract

In the adult skeleton, bone remodeling is required to replace damaged bone and functionally adapt bone mass and structure according to the mechanical requirements. It is regulated by multiple endocrine and paracrine factors, including hormones and growth factors, which interact in a coordinated manner. Because the response of bone to mechanical signals is dependent on functional estrogen receptor (ER) and Wnt/β-catenin signaling and is impaired in postmenopausal osteoporosis by estrogen deficiency, it is of paramount importance to elucidate the underlying mechanisms as a basis for the development of new strategies in the treatment of osteoporosis. The present study aimed to investigate the effectiveness of the activation of the ligand-dependent ER and the Wnt/β-catenin signal transduction pathways on mechanically induced bone formation using ovariectomized mice as a model of postmenopausal bone loss. We demonstrated that both pathways interact in the regulation of bone mass adaption in response to mechanical loading and that the activation of Wnt/β-catenin signaling considerably increased mechanically induced bone formation, whereas the effects of estrogen treatment strictly depended on the estrogen status in the mice.

## 1. Introduction

In the adult skeleton, adaptive bone remodeling is required to replace damaged bone and functionally adapt bone mass and structure according to the mechanical requirements throughout life. [1,2,3]. Mechanoadaption is dependent on multiple orchestrated endocrine hormones and paracrine growth factors with Wnt/β-catenin and estrogen receptor (ER) signalling as key players.

The important role of estrogen in functional bone mass adaptation is clearly apparent both in women with postmenopausal osteoporosis and in men with age-related bone loss, where the decline of estrogen decreases bone mass although functional loading occurs [1,4]. It has already been shown that estrogen receptor (ER) signaling is involved in mechanotransduction, mediating bone mass accrual [4,5,6]. ERα and ERβ, zinc finger-containing transcription factors and members of the nuclear receptor superfamily, regulate bone formation and remodeling through ligand-dependent and ligand-independent nuclear (genomic) mechanisms or membrane-associated (non-genomic) mechanism [4,7]. Nuclear mechanisms comprise classical and non-classical pathways that have direct binding to DNA at the estrogen response elements (ERE) or binding of (un)liganded ER to other DNA-bound transcription factors and, subsequently, indirect DNA binding in protein-protein complexes, including e.g., ER/specificity protein (SP) and ER/activating protein (AP)1 [7]. Non-genomic extranuclear effects of estrogen are mediated by classical ERs localized close to the plasma membrane and/or by the G-protein-coupled estrogen receptor 1 (GPER, GPR30), which is mainly found intracellularly [7,8]. Membrane-associated receptor activation involves rapid signaling through non-receptor tyrosine kinases of the Src family and the PI3K-Akt and MAPK pathways as well as second messenger molecules, including cAMP and calcium. Estrogens directly increase osteocyte and osteoblast lifespan by inhibiting their apoptosis and regulate osteoclast formation, activity and survival. In addition, indirect effects on osteoclasts are mediated via ER-related effects by osteoblasts, T cells and B cells. [9]. Thereby, estrogens decrease bone resorption and maintains bone formation. ERα is required for mechanically induced cortical bone formation in female mice and ERα acts in this process in a ligand-independent manner, involving activation function AF1, but not AF2 [10,11].

The impact of canonical Wnt/β-catenin signaling on bone formation and mechanoadaptation has been demonstrated, e.g., by the association of loss of function and gain of function mutations in the Wnt coreceptor low-density lipoprotein receptor related protein 5 (LRP5), which are associated with osteoporosis or high bone mass, respectively, in mice and humans [12,13,14]. Binding of Wnt glycoproteins, including Wnt3a to Frizzled (Fzd)-family/LRP5/6 receptor complexes, activates a cascade of signaling events that inhibits glycogen synthase kinase-3β (Gsk-3β) dependent phosphorylation of β-catenin and constitutive proteasomal β-catenin degradation [15]. The activated hypo-phosphorylated form of β-catenin translocates into the nucleus, where it binds to members of the transcription factors lymphoid enhancer binding factor-1 (Lef1) and T-cell factor (Tcf), regulating expression of genes important for proliferation, differentiation and apoptosis as well as functionality of bone cells. It has been demonstrated that Lrp5 and β-catenin function have a significant influence on the responsiveness of cortical bone to loading [16,17]. There is strong evidence for molecular crosstalk between ER and Wnt signaling in preosteoblastic cells involving, e.g., FHL1 as a molecular target [18]. Moreover, ERα signaling is requisite for the efficiency of Wnt/β-catenin signaling in the early response of osteoblastic cells to mechanical strain [19]. Both signal transduction pathways interact in the anabolic response to mechanical stimuli, thereby regulating the proliferation and expression of early mechanosensitive genes [20,21].

Wnt/β-catenin signaling is a key signaling cascade for osteogenic cell differentiation and bone formation, while ER signaling is a strong modulator of bone mass maintenance and estrogen deficiency is still one of the most prominent risk factors for osteoporosis. As discussed above, the response of bone to mechanical signals is dependent on both functional ER and Wnt/β-catenin signaling, but the net effects on mechanotransduction are dependent on the estrogen status of vertebrates (e.g., ovariectomy) at least in mice, and may also be altered in postmenopausal osteoporosis in humans [4,11,16]. The present study aimed to further dissect the molecular mechanisms of the impact of either pathway (ligand-dependent ER and Wnt/β-catenin signaling) on mechanotransduction in bone cells and their mutual molecular interactions using ovariectomised mice as a model of postmenopausal bone loss under estrogen-deficiency.

## 2. Results

### 2.1. Effects of Ovariectomy (OVX) and Estradiol Treatment

As expected, OVX reduced uterine weight compared to non-ovariectomized (Sham) mice (Table 1). Estradiol supplementation 4 weeks after ovariectomy for 2 weeks increased serum estradiol levels in OVX mice. Additionally, uterus atrophy was reversed 2 weeks after estrogen replacement.

As a consequence of OVX, cortical thickness (Ct.Th) was significantly decreased, and the marrow area (Ma.Ar) was significantly increased in OVX mice compared with Sham mice (Table 1). As expected, estrogen application decreased the Ma.Ar in OVX mice compared to OVX mice without estradiol treatment.

In the trabecular bone compartment, estrogen replacement increased the bone mass as confirmed by an elevated BV/TV, Tb.N and trabecular thickness (Tb.Th) in OVX mice compared to OVX mice without estradiol replacement. Accordingly, the Tb.Sp was reduced in estradiol-treated OVX mice compared with OVX mice without estradiol treatment.

### 2.2. Mechanical Loading Increases Cortical Bone Formation and Activates ER or β-Catenin Signaling in Ovariectomized Mice

Dynamic histomorphometry was performed to analyze the effects of estradiol and Gsk-3β inhibitor treatment on the bone response to mechanical loading in the Sham and OVX groups.

**Figure 1:**
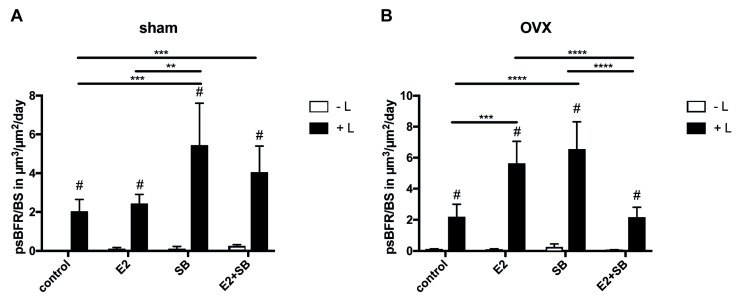
Sensitizing effect of estrogen receptor and Wnt signaling on mechanically induced bone formation in ovariectomized mice. The ulnae of Sham operated mice (Sham) (**A**) and ovariectomized mice (OVX) (**B**) were not subjected (☐ − L) or subjected to physiological loading (■ + L; peak surface strains –2500 μstrain) for 5 days/week for 2 weeks. The periosteal bone formation rate per bone surface (pBFR/BS) was assessed by dynamic histomorphometry. Bars represent mean ±SD (*n* = 4–6/group, ** *p* ≤ 0.01, *** *p* ≤ 0.001, **** *p* ≤ 0.0001 (E2: 17β-estradiol, SB: SB415286; −L: unloaded, +L: loaded), # value *p* ≤ 0.05 for loaded vs. unloaded ulna.

Mechanical loading increased the PsBFR/BS in both Sham and OVX animals (Figure 1A,B). Combined mechanical loading and estradiol treatment did not result in a significant difference in periosteal bone formation in the Sham group compared to the loaded Sham group without estradiol treatment (Figure 1A). By contrast, combined mechanical loading and Gsk-3β inhibitor application resulted in a significant increase of the PsBFR/BS in the Sham group compared with both the loaded Sham group without Gsk-3β inhibitor treatment and the loaded Sham group with estradiol treatment. Mechanical loading together with estradiol and additional Gsk-3β inhibitor treatment resulted in a significant increase of the PsBFR/BS, but bone formation tended to be reduced compared with the increase in the loaded group treated with the Gsk-3β inhibitor alone (Figure 1A).

In the OVX group, mechanical loading increased the PsBFR/BS (Figure 1B) similarly compared with the loaded Sham group. The Gsk-3β inhibitor as well as estrogen treatment had sensitizing effects on the mechanically induced anabolic response to mechanical loading. This sensitizing effect was significantly decreased when all treatments were combined in OVX mice (Figure 1B).

### 2.3. ERα and Wnt/β-Catenin Signaling Interact in the Mechanically Induced Response of Preosteoblastic Cells

**Figure 2:**
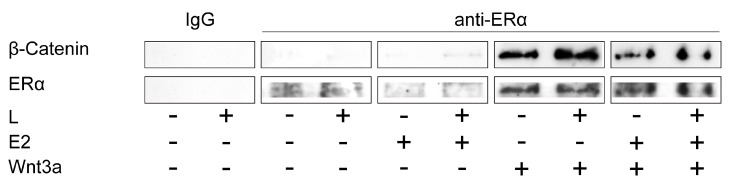
ER and Wnt/β-catenin signaling interact in the mechanically induced response of preosteoblastic cells. Preosteoblastic MC3T3-E1 cells were seeded in collagen-coated silicone dishes and mechanically loaded (2%, 1 Hz, 1800 cycles). Unloaded MC3T3-E1 cells were used as a control. 17β-Estradiol (E2, 10 nM) and/or Wnt3a (3 nM) were added to serum-starved MC3T3-E1 cell cultures for 3 h to activate ER and Wnt signaling, respectively, before loading (L) was applied. Western blotting was performed after immunoprecipitation with ERα antibodies. ERα pulled down β-catenin in response to Wnt/β-catenin signaling activation and mechanical loading. Representative immunoblot analyzed by using antibodies for unphosphorylated ERα and β-catenin.

To support an in vivo interaction of ERα and the Wnt/β-catenin signaling key component β-catenin in the mechanically induced response in preosteoblastic cells, we performed a co-immunoprecipitation assay using antibodies to ERα and the physiological Wnt/β-catenin signaling activator Wnt3a. The results indicated the physical association of ERα with β-catenin that was activated by estrogen and Wnt3a and, additionally, together with mechanical loading, demonstrated the cross-talk of both pathways in the mechanically induced response (Figure 2).

### 2.4. Mechanical Loading Increases Wnt and ERα Target Protein Expression and Activates ERα or β-Catenin Signaling in Preosteoblastic Cells

To determine whether the effects of mechanical loading and the activation of β-catenin and ER signaling in vivo may result from direct effects of the physical stimulus and the pharmaceutical agents on preosteoblastic cells, we studied the response of MC3T3-E1 cells to cyclic stretching in the presence of the Gsk-3β inhibitor and estrogen.

**Figure 3:**
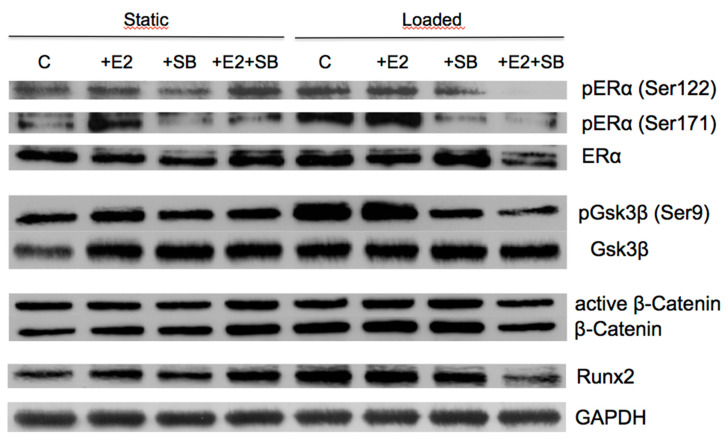
ER and Wnt/β-catenin signaling activation modulates mechanically induced gene expression in preosteblastic cells. Preosteoblastic MC3T3-E1 cells were seeded in collagen-coated silicone dishes and mechanically loaded (2%, 1 Hz, 1800 cycles). Unloaded MC3T3-E1 cells were used as a control. 17β-Estradiol (E2, 10 nM) and/or SB 415,286 (SB, 5 μM) were added to serum-starved MC3T3-E1 cell cultures for 3 h to activate ER and Wnt signaling, respectively, before loading was applied. Western blotting was performed using cell lysates isolated directly after loading. Representative immunoblot analyzed using antibodies for phospho-ERα (Ser122), phospho-ERα (Ser171), unphosphorylated ERα, phospho-glycogen synthase kinase (pGSK) 3β (Ser9), unphosphorylated GSK3β, unphosphorylated active β-catenin, total β-catenin and Runx2 as well as for GAPDH as control.

Treatment of the cells with estrogen resulted in ERα phosphorylation of at Ser122 and Ser171, which are the main phosphorylation sites in the AF1 region of ERα in response to estrogen [22] (Figure 3).

Estrogen led to an increase of phosphorylated Gsk3β at Ser9, which is known to inhibit Gsk3β activity, thereby activating β-catenin signaling [15]. Expression of both active β-catenin and total β-catenin was increased after estrogen treatment and additionally with SB415286. As expected, mechanical loading led to an increase of phosphorylated ERα and Gsk3β as well as an increase of β-catenin expression. In agreement with these observations, the expression of the ERα and β-catenin target protein Runx2 was increased in response to mechanical loading and estrogen or SB415286 treatment. Interestingly, mechanical loading and simultaneous activation of both ER and β-catenin signaling resulted in a decrease of phosphorylated ERα and Gsk3β as well as in a decrease of β-catenin. Consistent with these results, the level of the ERα and β-catenin target protein Runx2 decreased.

## 3. Discussion

In the present study, we demonstrated that the activation of Wnt/β-catenin signaling considerably increased mechanically induced bone formation, whereas the effects of estrogen treatment strictly depended on the estrogen status in the mice.

In our study, we used OVX mice as a widely accepted experimental model of postmenopausal bone loss [23]. The effects of mechanical loading on bone formation did not differ between the OVX and non-OVX groups. These results are in agreement with previous findings, indicating that the anabolic response of cortical bone to mechanical loading is independent of ovary-derived estrogen [24,25] and does not require the ligand-binding domain of ERα [11].

Furthermore, we demonstrated that estrogen replacement did not affect mechanically induced periosteal bone formation in non-OVX mice. However, in OVX mice, estrogen together with mechanical loading increased periosteal bone formation synergistically. These results imply that the mechano-sensitizing effect strictly depends on the estrogen status in mice. This observation is supported by a previous study reporting a synergistic interaction between mechanical loading and estrogen on osteogenesis, that is, the proliferation and matrix synthesis of periosteal osteoblasts using the ulnae of female rats [26]. Studies using genetically modified mouse models with selective or global ERα deletion provided evidence that ERα expression in osteoblast progenitors is required for periosteal bone apposition and the osteogenic response to mechanical loading, but independently from the activation by estrogen [10,11,27]. Furthermore, ERα expression and signaling have been shown to be crucial for vibration-induced bone formation in fracture healing [28], and ligand-independent ERα signaling seems to be responsible for the vibration-induced positive effects on osteoblasts in vitro [29,30]. In addition, ERα expression and activity were enhanced by estrogen and mechanical strain in osteoblastic cells in vitro [31,32]. The ERα expression levels in osteoblasts and osteocytes were shown to be higher in cortical than in cancellous bone and to be increased in the new bone formed at the periosteal surface after mechanical loading in vivo [33]. These findings suggest that the considerable increase of load-induced bone formation at the periosteal bone surface that we observed in the OVX mice after estrogen replacement may result, at least in part, from an increased function of mature osteoblasts mediated via enhanced ERα expression and/or activity. Indeed, it has been supposed that the failure of bone adaption to mechanical load in postmenopausal osteoporosis may be caused by reduced ERα expression because of estrogen decline [34].

Our results demonstrated that canonical Wnt signaling activation in mice by SB415286 synergistically enhanced mechanically induced periosteal bone formation in both non-OVX and OVX mice. Previous studies showed that canonical Wnt signaling promotes the progression of osteoblast progenitor cells to bone producing osteoblasts [35] and that unliganded ERα in these progenitor cells is able to potentiate canonical Wnt signaling, thereby promoting the proliferation and differentiation of periosteal osteoprogenitor cells [27]. Therefore, the increased periosteal bone accrual observed after SB415286 treatment in the present study might be due to enhanced proliferation and differentiation of osteoblast progenitor cells.

Notably, the mechano-sensitizing effect of SB415286 was abolished by additional estrogen treatment, particularly in OVX mice, suggesting an interaction of Wnt/β-catenin and estrogen signaling in the response of bone to mechanical load. It was demonstrated that Wnt3a-mediated proliferative and osteoblastic effects were blunted in periosteal progenitor cells from mice with specific ERα deletion in osteoblast precursor cells [27]. Interestingly, 17β-estradiol did not affect basal or Wnt3-induced proliferation of wildtype cells but rather attenuated Wnt3a-induced alkaline phosphatase (AP) activity, indicating that the effects mediated by ERα could be independent or even opposite to those of its ligand. In agreement with this, 17β-estradiol attenuated Wnt3a-stimulated AP activity in murine myoblastic C2C12 cells but had no effect on Tcf-mediated transcription. These observations are consistent with the results of our study showing decreased bone formation after simultaneous treatment with 17β-estradiol, SB415286 and mechanical loading.

To determine whether our in vivo results are caused by direct effects of estrogen, SB415286 and mechanical loading on osteoblast progenitor cells, we investigated the effects of loading and reagents in vitro using preosteoblastic MC3T3-E1 cells [36]. As expected, mechanical loading and estrogen or the Gsk3β inhibitor treatment resulted in upregulated expression of the transcription factor Runx2. Runx2 is indispensable for osteoblast differentiation and for the function of mature osteoblasts, including synthesis of structural bone matrix proteins [37] and is regulated by mechanical stimuli in osteoblastic cells [38]. Consistent with Runx2 upregulation, we observed increased ERα phosphorylation at Ser122 and Ser171 after estrogen treatment and mechanical loading alone. Ser 122 and Ser 171, which in human correspond to Ser 118 and Ser 167, respectively, are the main phosphorylation sites in the AF1 region of ERα in response to estrogen [22]. AF1 of ERα has been shown to be involved in mechanically induced cortical bone formation in female mice in a ligand-independent manner, but without AF2 [10,11]

Moreover, as expected, estrogen treatment alone and together with mechanical stimulation increased Gsk3β phosphorylation at Ser9, which is required for the inhibition of Gsk3β, which prevents β-catenin phosphorylation and its proteosomal degradation, thus enabling β-catenin-inducible transcription [15]. Gsk3β phosphorylation at Ser9 has already been demonstrated in vitro on estrogen treatment or mechanical loading [19,39]. Mechanical loading and additional treatment with the Gsk3β inhibitor resulted in a decreased ERα phosphorylation level at Ser122 and Ser171 in comparison with the phosphorylation level detected after treatment with mechanical load alone. ERα transcriptional activity was demonstrated to be decreased through inhibition of Gsk3 by SB415286 [40]. Additionally, mechanical loading and combined with the Gsk3β inhibitor reduced the level of Gsk3β phosphorylation at Ser9 compared to the phoshorylation level that was detected after the application of the mechanical load alone. Activating phosphorylation of both Gsk3β and ERα was further decreased in response to the treatment with the Gsk3β inhibitor and estrogen together with mechanical load. In agreement with this, we detected reduced active β-catenin and Runx2 expression.

In summary, we demonstrated a sensitizing effect of estrogen on mechanically induced bone formation in OVX mice, but not in non-OVX mice, underscoring the important role of the estrogen status in the anabolic response to load. Canonical Wnt signaling activation by Gsk3β inhibition and mechanical loading acted, when combined, synergistically to induce bone formation in both non-OVX and OVX mice. Additional estrogen treatment abolished the positive effects of canonical Wnt signaling activation on load-induced bone apposition. Our study suggests that the osteogenic response of bone to mechanical loading, at least in mice, is dependent on a well-coordinated activity of signaling pathways. These results may have important implications for the treatment of osteoporotic patients. Clinical studies should be undertaken to investigate whether exercise or external mechanical stimulation, for example, a vibration therapy, increased the osteoanabolic effect of Wnt-targeting drugs also in humans.

## 4. Materials and Methods

### 4.1. Mice

Female CD1 mice (Jackson Laboratory, Bar Harbor, ME, USA) were used for the study. The mean body weight was 26.8 g ± 2.9 g. Mice were housed under a regular light/dark cycle and were fed a standard rodent diet until aged 10 weeks. Subsequently, they were fed a phytoestrogen-reduced diet (R/M-H low phytoestrogen, ssniff GmbH, Soest, Germany). All experimental procedures were performed according to the national and international regulations for the care and the use of laboratory animals and were approved by the National Ethics Committee (Germany, Regierungspräsidium Tübingen, Reg.Nr. 1019).

### 4.2. Ex Vivo Calibration of the Ulna Loading Regimen and Mechanical Loading In Vivo

We used the well-accepted ulna-loading model ulna loading [41] to study the functional adaption of the cortical bone by applying dynamic axial compression to the ulna. Firstly, the loading regimen was calibrated ex vivo using four ulnae of 16-week-old female CD1 mice. Strain gauges (HBM, Darmstadt, Germany) were applied to the medial surface at the midshaft of the right ulna of dead mice, and the ulna was positioned in the loading apparatus established according to the study of Lee et al. [41]. The loads required to produce peak surface strains of −2000 to −3000 µstrain correlating with strain magnitudes generated during normal locomotion [42] were recorded from the ulna midshaft.

**Scheme 1:**
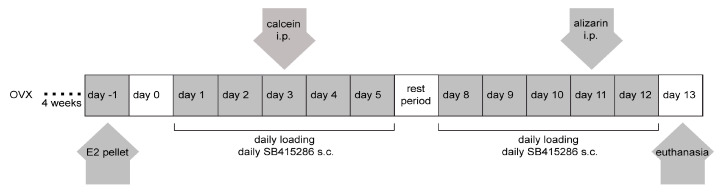
Treatment regimen. Abbr.: Ovarectomy (OVX), estradiol (E2), intraperitoneal (i.p.), subcutaneous (s.c).

In vivo loading was performed 4 weeks after ovariectomy of the mice when aged 16 weeks (Scheme 1). The right ulna was loaded for 120 cycles, using a 2 Hz trapezoidal waveform and an axial compressive peak load of 2.5 N, correlating to a peak surface strain of −2500 µstrain, 5 days per week for 2 weeks. The left ulna served as an internal non-loaded control. During loading, mice were anesthetized by inhalation of 2% isoflurane. Mice were euthanised 24 h after the last mechanical loading by carbon dioxide inhalation.

### 4.3. Ovariectomy and Estradiol and Gsk-3β Inhibitor Treatments

When aged 12 weeks, mice underwent bilateral OVX or Sham operation (non-OVX) by dorsal midline incision under 2% isoflurane inhalation anesthesia. Analgesia was ensured by oral Tramal administration (25 mg/L drinking water) until day 3 post surgery. To avoid wound infection, the mice received 45 µg/g clindamycin-2-dihydrogenphosphate by subcutaneous injection directly before surgery. At 4 weeks after surgery and 2 days before mechanical loading was applied, a subcutaneous estradiol pellet (0.18 mg 17β-estradiol per 60-day release pellet (Innovative Research of America, Sarasota, FL, USA) or a placebo pellet was applied subcutaneously in the neck (Scheme 1). At 6 weeks after surgery, blood samples were collected for serum estradiol analysis by ELISA (Estradiol ELISA, Calbiotech, El Cajon, CA, USA), and uteri were removed and weighed.

To activate Wnt/β-catenin signaling, 1 mg/kg body weight of the GSK-3β inhibitor SB415286 (3-(3-chloro-4-hydroxyphenylamino)-4-(2-nitrophenyl)-1H-pyrrole-2,5-dione, Tocris Bioscience, Bristol, UK) was subcutaneously injected daily during the time period of mechanical loading. Control mice received vehicle solution.

### 4.4. Histomorphometry and Bone Microstructure Measurements

For histomorphometry, each mouse was injected intraperitoneally with calcein green (0.03 µg/g body weight, Sigma Aldrich, Munich, Germany) on the 3rd day and with alizarin red (0.05 µg/g body weight) on the 11th day of mechanical loading. The right and left ulna of each mouse were cleaned of soft tissue, fixed in 4% paraformaldehyde, dehydrated in ethanol, and embedded in methyl methacrylate. Ten serial cross-sections of the undecalcified ulnae perpendicularly to the long axis were cut with 10 µm thickness at 2 mm distal from the midshaft. Sections were analyzed using the inverted microscope DMI6000 B (Leica, Wetzlar, Germany) with a double filter for green/red fluorescence and Leica MMAF software (Leica MetaMorphAF 1.4.0, Heerbrugg, Switzerland). Histomorphometric analysis was performed according to the guidelines of the American Society for Bone and Mineral Research (ASBMR) [43,44]. Bone surface (BS), single (sLS/BS) and double (dLS/BS) labeled bone surface, mineralizing surface (MS), inter-label thickness (Ir.L.Th), and mineral apposition rate (MAR = Ir.L.Th/Ir.L.t) were measured and calculated with Ir.L.t = 8 days. The PsBFR/BS bone formation rate with BS as the referent was calculated as MAR × MS/BS × d (μm^3^/μm^2^/day).

Three-dimensional bone reconstructions were generated by microcomputed tomography (Skyscan 1172, Skyscan, Belgium) at a spatial resolution of 8 µm [45]. A region (160 μm) at 2 mm distal from the midshaft of the right and left ulna was analyzed for Ma.Ar and C.Th, to determine the influence of ovariectomy, mechanical loading, estradiol, and the GSK-3β inhibitor on cortical bone. The trabecular bone of L5 vertebra was analyzed for Tb.Th, Tb.N, Tb.Sp, bone volume (BV), and tissue volume (TV), and BV/TV was calculated to determine the influence of ovariectomy and estradiol treatment on trabecular bone.

### 4.5. Mechanical Loading In Vitro

Preosteoblastic MC3T3-E1 cells were seeded at a density of 10,000 cells/cm^2^ in α-minimal essential medium (α-MEM, Biochrom, Berlin, Germany) supplemented with 10% fetal calf serum (PAA Laboratories, Coelbe, Germany), 1% glutamine (Biochrom) and 1% penicillin/streptomycin (Gibco, Life Technologies, Darmstadt, Germany) and cultured in 5% CO_2_ at 37 °C and saturation humidity in precoated (5 μg/cm^2^ collagen type I solution; BD Biosciences, Bedford, USA) flexible silicone dishes. Mechanical loading of cells was performed on day 5 of cultivation by homogenous cyclic stretching as described previously [46]. We applied sinusoidal strain at 2% and a frequency of 1 Hz for 1800 cycles (equivalent to 30 min). Control dishes were prepared in parallel using an identical procedure, but no load was applied. 17β-Estradiol (10 nM, Sigma-Aldrich, Taufkirchen, Germany) or SB 415,286 (5 μM) were added to serum-starved preosteoblastic cell cultures 3 h before loading was applied. Control dishes were prepared in parallel using the vehicle, otherwise all the same experimental conditions were used.

### 4.6. Co-Immunoprecipitation

MC3T3-E1 cells were cultivated for 5 days. Wnt3a (3 nM) and estrogen (10 nM) were added in serum-starved α-MEM with 0.25% bovine serum albumin 3 h before loading was applied.

Immunoprecipitation was performed as described previously [45]. In brief, cells were washed twice with ice-cold phosphate-buffered saline and immunoprecipitation lysis buffer (20 mM Tris-HCl pH 7,5, 150 mM NaCl, 1 mM EDTA, 10 μM/ml 100× Halt protease, and phosphatase inhibitor cocktail (Thermo Fisher Scientific, Waltham, MA, USA). Protein A-Sepharose (Sigma), linked to either goat immunoglobulin G or ERα antibody (Abcam, Cambridge, MA, USA), was added to the whole cell lysis supernatant after centrifugation at 12000× g and incubated overnight at 4 °C with gentle agitation. Protein complexes were dissociated by resuspension in 3× SDS sample buffer, incubated for 5 min at 37 °C and used for western blotting.

### 4.7. Western Blot Analysis

Western blotting was performed as described previously [46]. Aliquots of 15 μg cellular lysate protein were resolved by SDS-polyacrylamide gel electrophoresis (10% resolving gel) and subsequently transferred to a nitrocellulose membrane (AmerSham Biosciences, USA). Membranes were incubated with phospho-ERα (Ser122; Biorbyt, Cambridge, UK), phospho-ERα (Ser167; Abcam, UK), ERα (Abcam), phospho-GSK-3β (Ser9; Cell Signaling, Danvers, MA, USA), GSK-3β (Cell Signaling), active-β-catenin (Millipore, Molsheim, France), β-catenin (Millipore), and RUNX2 (Cell Signaling) antibodies overnight at 4 °C, respectively. To demonstrate equivalency of protein loading, a specific GAPDH antibody (Cell Signaling) was used. The membranes were incubated with horseradish peroxidase-conjugated goat anti-rabbit, rabbit anti-sheep or goat anti-mouse secondary antibody (Cell Signaling) for 1 h at room temperature. For the enhanced chemiluminescence reaction, immunoblots were developed in SuperSignal West Pico Chemilumemescent Substrate (Perbio Science, Pierce, Bonn, Germany), and the respective phosphoproteins or total proteins were visualized using the Fusion Molecular Imaging System (Vilber Lourmat, Eberhardzell, Germany).

### 4.8. Statistical Analysis

Each experimental group included 4–6 mice (*n* = 4–6). SPSS software (SPSS GmbH, Version 18, München, Germany) was used for statistical analysis. Sham (non-OVX), OVX and OVX + E2 treated groups were statistically analyzed using two-way ANOVA with Bonferroni post hoc test. Untreated, estradiol-treated, SB415286-treated, and estradiol together with SB415286 treated non-loaded and loaded groups were analyzed using two-way ANOVA with Turkey post hoc correction. Data obtained were analyzed for significance (value *p* ≤ 0.05). The results were presented as mean value ± standard deviation of the mean (SD).

## Figures and Tables

**Table 1:** Influence of estrogen supplementation on estrogen (E2) level, uterus weight and trabecular as well as cortical properties of the vertebral bodies (L5) and ulnae of CD1 mice 6 weeks after ovariectomy.

Parameter.	Sham	OVX	OVX+E2
**Estrogen Level (Serum)**			
Estrogen in pg/mL	13.7 ± 4.1	9.9 ± 1.2	78.7 ± 13.6 ^bc^
**Uterus**			
Uterus weight in mg	225.2 ± 80.2	66.7 ± 31.8 ^a^	283.7 ± 41.5 ^c^
**Trabecular Bone (L5)**			
BV/TV in %	13.9 ± 1.1	11.6 ± 1.3	16.8 ± 2.4 ^bc^
Tb.Th in mm	0.059 ± 0.002	0.058 ± 0.002	0.062 ± 0.003 ^c^
Tb.N in 1/mm	2.4 ± 0.2	2.0 ± 0.2	2.7 ± 0.4 ^c^
Tb.Sp in mm	0.26 ± 0.02	0.30 ± 0.02	0.25 ± 0.02 ^c^
**Cortical Bone (Ulna)**			
Ct.Th in mm	0.180 ± 0.007	0.168 ± 0.007 ^a^	0.172 ± 0.004
Ma.Ar in mm^2^	0.028 ± 0.002	0.037 ± 0.001 ^a^	0.032 ± 0.002 ^bc^

*n* = 6 mice/group; data are presented as mean ± SD. ^a^ *p* ≤ 0.05 OVX vs. Sham; ^b^ *p* ≤ 0.05 OVX+E2 vs. Sham; ^c^ *p* ≤ 0.05 OVX+E2 vs. OVX.
